# Editorial: Cell polarity: Trafficking and regulatory events that determine cell asymmetry

**DOI:** 10.3389/fcell.2023.1119485

**Published:** 2023-01-12

**Authors:** Andrés E. Zucchetti, James R. Goldenring, M. Cecilia Larocca

**Affiliations:** ^1^ Institut Curie, PSL Research University, INSERM U932, Paris, France; ^2^ Epithelial Biology Center and Department of Cell & Developmental Biology, Vanderbilt University School of Medicine, Nashville, TN, United States; ^3^ Instituto de Fisiología Experimental, Consejo Nacional de Investigaciones Científicas y Técnicas, Facultad de Ciencias Bioquímicas y Farmacéuticas, Universidad Nacional de Rosario, Rosario, Argentina

**Keywords:** primary cilia, sonic hedgehog, cofilin, neuronal polarity, epithelial polarity, phosphoinositides

**FIGURE 1:**
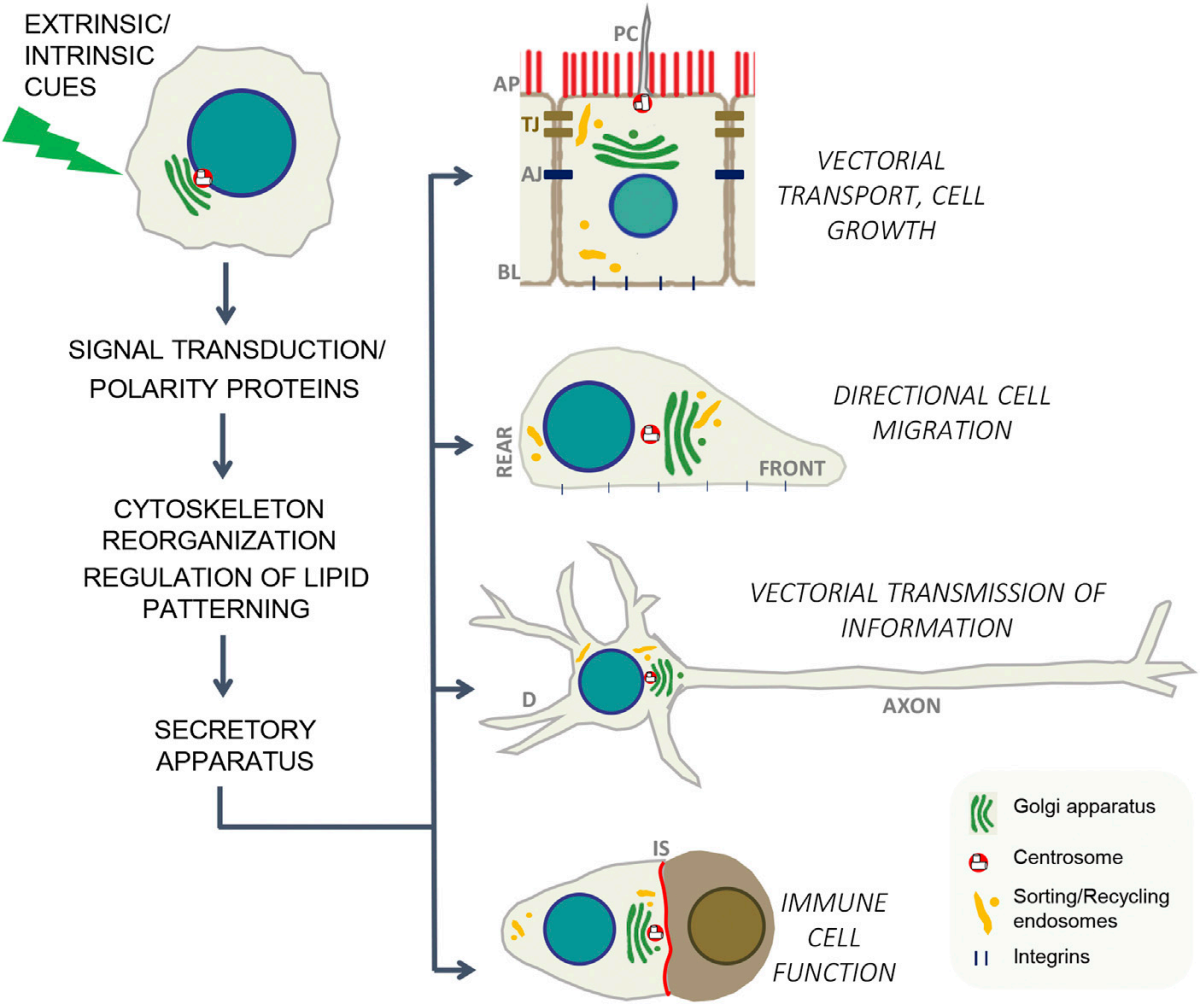
In response to intrinsic or external cues, each cell type has adapted a common set of evolutionarily conserved mechanisms to generate polarity. Those mechanisms include: localized assembly of signaling/polarity complexes, cytoskeleton remodeling, lipid re-patterning and targeted vesicle delivery to specific cell domains (Nelson, 2003; Mellman and Nelson, 2008). AP, apical; BL, basolateral; PC, primary cilium, TJ, tight junctions; AJ, adherent junctions; D, dendrites; IS, immune synapse.

Cell polarity refers to the asymmetric distribution of cellular components along defined axes of the cell, which constitutes a fundamental property of most cells, from single-cell organisms to cells in multicellular invertebrates and vertebrates (Nelson, 2003). The ability of cells to generate a specific spatially biased biochemical and morphological organization in response to distinct extracellular or intracellular cues is critical for development and for specialized cell functions, including directional cell migration, phagocytosis, epithelial secretion and absorption, immune response, and transmission and transduction of information by the nervous system (Bryant and Mostov, 2008). Furthermore, cell polarity has a main role in the control of epithelial cell growth, what is underscored by the relationship between tumor suppressors and apico-basal polarity and the fact that loss of polarity is an early hallmark of malignancy arising from epithelial tissues, which constitute the major cause of fatal cancer in adults (Wodarz A and Näthke, 2007). The attainment of the precise, time and spatially organized cell asymmetry in response to extracellular and/or intracellular cues is triggered by different signals in each particular system, but it is orchestrated by a common mechanistic pattern that comprise activation of compartmentalized signaling pathways, major rearrangements of the cytoskeleton, regulation of lipid landscape and polarized membrane trafficking (Figure 1). The present Research Topic contains two review articles, two original research full articles and one brief research report addressing cellular mechanisms that determine cell polarity, which have impact on epithelial homeostasis, directional cell migration, phagocytosis and neuronal function.

Neuron polarity is a paradigmatic case of programmed cell asymmetry, morphologically expressed in the dendritic and the axonal domains (Figure 1), which can be more or less conspicuous depending on the neuron type. The article “Perspectives on Mechanisms Supporting Neuronal Polarity From Small Animals to Humans” reviews critical mechanisms controlling axo-dendritic specification, most of which were elucidated using experimental animal models, highlighting Research Topic that should be considered in new experimental systems, such as neurons differentiated from human induced pluripotent stem cells and human brain organoids Overall, the authors collect evidence supporting the concept that molecular switches driving neuronal polarity are conserved across cell types, tissues and species, and point out biophysical, genetic and epigenetic factors that contribute to shaping neurons, which remain understudied.

Historically, the epithelial polarity has been primarily described in relation to the localization and function of protein “polarity complexes”. However, a critical and foundational role is emerging for plasma membrane lipids, and in particular phosphoinositide species. The review article “Membrane Lipids in Epithelial Polarity: Sorting out the PIPs” written by Bugda Gwilt and Thiagarajah overviews the evidence for a primary role for membrane lipids in the generation of epithelial polarity. The authors broadly describe the evidence for a primary role for membrane lipids in the generation of epithelial polarity, concentrating principally, but not exclusively, on intestinal epithelia. They particularly review the complex interplay and interchange that exists between different phosphoinositide species and describe how major membrane lipid constituents are generated, intersect with vesicular trafficking, and are localized to different membrane domains; focusing on key protein complexes involved in these processes. Finally, the article reviews the existing evidence for specific membrane lipid species as drivers of apico-basolateral polarity and highlights key areas requiring further research.

The primary cilium is an organelle present at the apical pole of every epithelial cell of simple polarity (Figure 1), which senses liquid flow, osmotic pressure and growth signals present at the luminal media, transducing them into intracellular cues that can regulate cell cycle and apico-basolateral epithelial polarity. The brief research report “Short-Chain Fatty Acid Butyrate Induces Cilia Formation and Potentiates the Effects of HDAC6 Inhibitors in Cholangiocarcinoma Cell” addresses the regulation of the ciliogenesis by butyrate, a metabolite produced by bacterial fermentation of fibers in the intestines (Pant et al., 2022). In the present article, the authors demonstrate that butyrate treatment rescues cilia formation in cholangiocarcinoma cells, which is positively linked with cell growth inhibition. The article also shows that butyrate and HDAC6 inhibitors elicit synergistic effects on halting cell growth, epithelial mesenchymal transition and migration in those cells, suggesting that lower doses, which would be innocuous to healthy cells, could be used to restore cell homeostasis in cholangiocarcinoma cells.

Hedgehog proteins are signaling determinants of differentiation and organogenesis, which contribute to tissue homeostasis and repair. Whether sonic hedgehog (Shh) proteins are secreted at the apical or at the basolateral pole of epithelial cells will condition Shh targets and, therefore, Shh function. The original research article “Sonic hedgehog is basolaterally sorted from the TGN and transcytosed to the apical domain involving Dispatched-1 at Rab11-ARE″ investigates trafficking factors that conditions the epithelial pole where Shh proteins are secreted (Sandoval et al., 2022) demonstrate that, in polarized MDCK cells, newly synthesized Shh traffics from the trans-Golgi network to the basolateral surface and then is transcytosed to the apical pole, where most of its secretion occurs. The article further shows that Shh membrane attachment through palmitoylation and cholesteroylation differentially contributes to this indirect trafficking pathway. Furthermore, it provides data suggesting that Dispatched-1 not only promotes basolateral Shh secretion, but also facilitates Shh transcytotic traffic through the Rab11-positive apical recycling endosomes. Overall, this article characterizes a complex trafficking pathway for Shh proteins, which offers multiple points for regulation to determine at which epithelial pole these signaling proteins are secreted.

Entamoeba histolytica infection requires a direct contact between the parasite and the host cell. Many molecular components of the parasite are recruited at the contact zone to induce phagocytosis and, in a last step, killing of the host cell. Although it has been well established that phagocytic cup formation is conditioned by Entamoeba actin cytoskeleton reorganization at specific sites at the contact zone, the molecular mechanisms that regulate this process are still unknown. Cofilins are members of the actin-depolymerizing factor/cofilin family of proteins, which regulates actin dynamics in response to cellular signaling and mechanical cues. The original research article “Unravelling the Biology of EhActo as the First Cofilin From Entamoeba histolytica” by identifies Entamoeba histolytica actophorin (EhActo) as the first true cofilin from Entamoeba, which actively severs actin filaments. Studies of NMR structure modeling show that EhActo contains an actin depolymerizing factor (ADF)-homology domain. Comparison of sequences with other cofilins showed that EhActo lacks Serine at the first five amino acids residues, unraveling a unique mechanism of action for this protein. EhActo was also present in the phagocytic cups of the amoebic cells during erythrophagocytosis, strongly suggesting a role in the infection of the pathogen. Indeed, the authors showed that EhActo interacts specifically with EhP3 (Entamoeba histolytica 14-3- 3 protein isoform 3), a protein previously described to play a role in Entamoeba phagocytosis. In summary, this article hints at an unexplored differential regulatory mechanism operational in a primitive pathogen.
